# Association of germline BRCA and homologous recombination deficiency with hematologic toxicity during platinum–taxane chemotherapy in ovarian cancer

**DOI:** 10.1007/s10147-026-03065-4

**Published:** 2026-05-27

**Authors:** Kosuke Yoshida, Hajime Araki, Yurika Yamada, Yoshiki Masahashi, Emiri Miyamoto, Komei Katayama, Mei Kubokawa, Kazumasa Mogi, Masato Yoshihara, Yukari Nagao, Satoshi Tamauchi, Akira Yokoi, Nobuhisa Yoshikawa, Kaoru Niimi, Hiroaki Kajiyama

**Affiliations:** 1 Department of Obstetrics and Gynecology, Nagoya University Graduate School of Medicine, Tsuruma-cho 65, Showa-ku, Nagoya, 466-8550 Japan; 2 Department of Medical Genomics Center, Nagoya University Hospital, Nagoya, Japan

**Keywords:** Ovarian cancer, Germline BRCA, Homologous recombination deficiency, Hematologic toxicity, Platinum–taxane chemotherapy

## Abstract

**Background:**

Germline *BRCA* (gBRCA) mutations and homologous recombination deficiency (HRD) are critical factors affecting treatment response; however, their association with hematologic toxicity remains controversial. This study assessed the interplay between these molecular features and hematologic adverse events during platinum–taxane chemotherapy.

**Methods:**

We retrospectively reviewed 62 patients with stage III–IV ovarian cancer between 2022 and 2024. After excluding 8 patients, 54 patients were stratified according to gBRCA and HRD status, and hematologic adverse events were evaluated for up to six treatment cycles. Moreover, meta-analysis was performed to integrate previously published data.

**Results:**

First, after excluding patients with uncertain gBRCA status, eight gBRCA mutation carriers were compared with 33 confirmed non-carriers. gBRCA mutation carriers tended to exhibit lower neutrophil counts during treatment; however, no statistically significant differences in hematologic toxicities were observed across treatment cycles. In the pooled meta-analysis, gBRCA mutation carriers demonstrated significantly increased odds of neutropenia (odds ratio [OR] 1.68, 95% confidence interval [CI] 1.16–2.44) and G-CSF use (OR 3.09, 95% CI 1.19–8.03), whereas no significant associations were observed for anemia, thrombocytopenia, dose delay, or dose reduction. Second, 23 HRD-positive patients and 15 HRD-negative patients were analyzed. HRD-positive patients did not demonstrate increased hematologic toxicity compared with HRD-negative patients. Although baseline hemoglobin levels were lower in HRD-positive patients, these differences preceded chemotherapy initiation.

**Conclusion:**

This study provides real-world evidence of a modestly increased risk of neutropenia and G-CSF use in gBRCA carriers during platinum–taxane chemotherapy; however, these differences did not appear to compromise treatment delivery.

**Supplementary Information:**

The online version contains supplementary material available at 10.1007/s10147-026-03065-4.

## Introduction

Ovarian cancer (OC) is a major cause of cancer-related death among women worldwide, resulting in approximately 200,000 deaths annually [1]. OC comprises several histological subtypes, with high-grade serous carcinoma (HGSC) being the most common one [2]. Accumulating evidence suggests that HGSC originates from the epithelium of the fallopian tube and rapidly disseminates to the ovary and peritoneal surfaces [3, 4]. Consequently, early diagnosis of HGSC is challenging, and the disease is usually diagnosed at an advanced stage, resulting in poor prognosis.

Recently, hereditary breast and ovarian cancer syndrome (HBOC) has attracted increasing attention. The cumulative risk of OC by age 80 is approximately 44% and 17% in carriers of *BRCA1* mutation and *BRCA2* mutation, respectively [5]. Therefore, some women with pathogenic germline mutations in *BRCA1* or *BRCA2* choose to undergo risk-reducing salpingo-oophorectomy, which has been shown to reduce OC risk and all-cause mortality [2, 6]. The introduction of poly(ADP-ribose) polymerase inhibitors (PARPis) has considerably changed the therapeutic landscape of OC. The standard treatment for advanced OC includes cytoreductive surgery and a combination of taxane- and platinum-based chemotherapy to achieve no residual disease [2]. Since the 2010s, anti-VEGF antibody therapy has also been introduced as both first-line treatment and maintenance therapy [2, 7]. More recently, PARPis have been widely used for maintenance therapy based on *BRCA1/2* mutation and homologous recombination deficiency (HRD) status [2]. A pivotal clinical trial demonstrated that maintenance therapy with bevacizumab plus olaparib significantly prolonged both progression-free survival (PFS) and overall survival (OS) in HRD-positive patients compared with placebo plus bevacizumab (PFS: hazard ratio [HR] 0.41, 95% confidence interval [CI] 0.32–0.54; OS: HR 0.62, 95% CI 0.45–0.85) [8]. In addition, niraparib monotherapy as maintenance treatment for newly diagnosed advanced OC significantly improved PFS compared with placebo (HR 0.62; 95% CI 0.50–0.76) [9]. Therefore, HBOC and PARPis have become increasingly important in OC management.

PARPis block the repair of single-strand DNA breaks by inhibiting PARP1 activity. Unrepaired single-strand breaks can lead to replication fork collapse during DNA replication, resulting in double-strand DNA breaks [10]. Normally, double-strand DNA breaks are repaired through homologous recombination (HR), a pathway in which *BRCA1* and *BRCA2* are critical. In tumor cells with biallelic BRCA1/2 inactivation, PARP inhibition leads to DNA damage accumulation and induces synthetic lethality [10]. In contrast, noncancerous somatic cells in germline *BRCA1/2* mutation carriers retain a single wild-type *BRCA*1/2 allele, resulting in a haploinsufficient state. However, the impact of *BRCA1/2* haploinsufficiency on hematopoietic cells remains unclear. Several clinical studies have investigated hematologic adverse events during OC treatment, with inconsistent results [11–15]. Therefore, in this study, we investigated the frequency of these events during first-line chemotherapy for OC according to *BRCA1/2* and HRD status, and further assessed the impact of *BRCA1/2* mutation status by integrating our findings with the results of previous studies.

## Patients and methods

### Patients and treatment

We retrospectively reviewed the medical records of patients with advanced OC (stage III–IV) who initiated treatment at our institution between 2022 and 2024. Treatment strategies were determined by several gynecologic oncologists according to the Japanese Guidelines for the Treatment of Ovarian Cancer (2020 edition). Based on the patient’s condition and the extent of the disease, either primary debulking surgery (PDS) or neoadjuvant chemotherapy followed by interval debulking surgery (NAC–IDS) was selected as the treatment approach. The standard first-line chemotherapy regimen consisted of paclitaxel (180 mg/m^2^) plus carboplatin (AUC 6, Calvert formula with renal function estimated by the Jaffé method; 0.2 mg/dL was added to enzymatic creatinine values to obtain Jaffé-equivalent levels). For patients with contraindications to paclitaxel, such as alcohol hypersensitivity, docetaxel (70 mg/m^2^) was used as an alternative. Bevacizumab was also administered to eligible patients.

Tumor somatic *BRCA1/2* (sBRCA1/2) mutations and HRD scores were assessed using the MyChoice^®^ CDx assay (Myriad Genetics, Salt Lake City, UT), and germline *BRCA* (gBRCA) testing was performed using the BRACAnalysis^®^ test (Myriad Genetics) following genetic counseling. Moreover, some patients received comprehensive genomic profiling after recurrence.

During treatment, patients underwent regular blood examinations for adverse events monitoring. Complete blood counts were routinely obtained between days 8 and 14 after chemotherapy administration, and these adverse events were evaluated according to the Common Terminology Criteria for Adverse Events (CTCAE), version 5.0. When grade 4 neutropenia was observed, granulocyte colony-stimulating factor (G-CSF) was administered, and pegfilgrastim was considered for the next cycle in some cases. Anemia and thrombocytopenia were managed with blood transfusions as appropriate.

This study was approved by the Ethics Committee of Nagoya University (Approval No. 2017-0053), and approval for the opt-out consent method was obtained. All procedures were conducted in accordance with relevant guidelines and regulations and with the ethical standards of the Declaration of Helsinki.

### Statistical analysis

All statistical analyses were performed using R (ver. 4.5.1) and RStudio (ver. 2025.09.0; RStudio, Boston, MA). Continuous variables were compared using Welch’s *t*-test, and categorical variables were compared using Fisher’s exact test. Given the multiple comparisons across six treatment cycles and multiple adverse event endpoints during the treatment period, *p*-values were adjusted using the Benjamini–Hochberg false discovery rate method. An adjusted *p*-value (*q*-value) < 0.05 was considered statistically significant. Patients who received pegfilgrastim (long-acting G-CSF) were excluded from the analyses of neutrophil counts and G-CSF use. Patients who underwent interval debulking surgery (IDS) or had their chemotherapy regimens changed due to disease progression were excluded from subsequent analyses.

For the meta-analysis, a systematic review was conducted in accordance with the PRISMA 2020 guidelines. PubMed and Scopus were searched from database inception to February 22, 2026, using predefined search terms related to ovarian cancer, germline BRCA mutation, platinum-containing chemotherapy, and hematologic toxicity. Detailed search strategies and the study selection process are provided in Supplementary Fig. S1. Predefined inclusion and exclusion criteria were established based on a PICO framework (Population: ovarian cancer patients; Exposure: germline BRCA mutation; Comparator: BRCA wild-type; Outcome: chemotherapy-related hematologic toxicity). After duplicate removal, titles and abstracts were screened, followed by full-text eligibility assessment. Pooled odds ratios (ORs) and 95% confidence intervals (CIs) were estimated using a random-effects model with DerSimonian–Laird estimation of between-study variance. Statistical heterogeneity was assessed using the *I*^2^ statistic. Separate meta-analyses were performed for each hematologic toxicity outcome. Risk of bias was evaluated using the Newcastle–Ottawa Scale (NOS), and the results are presented in Supplementary Table S1.

## Results

**Fig. 1:**
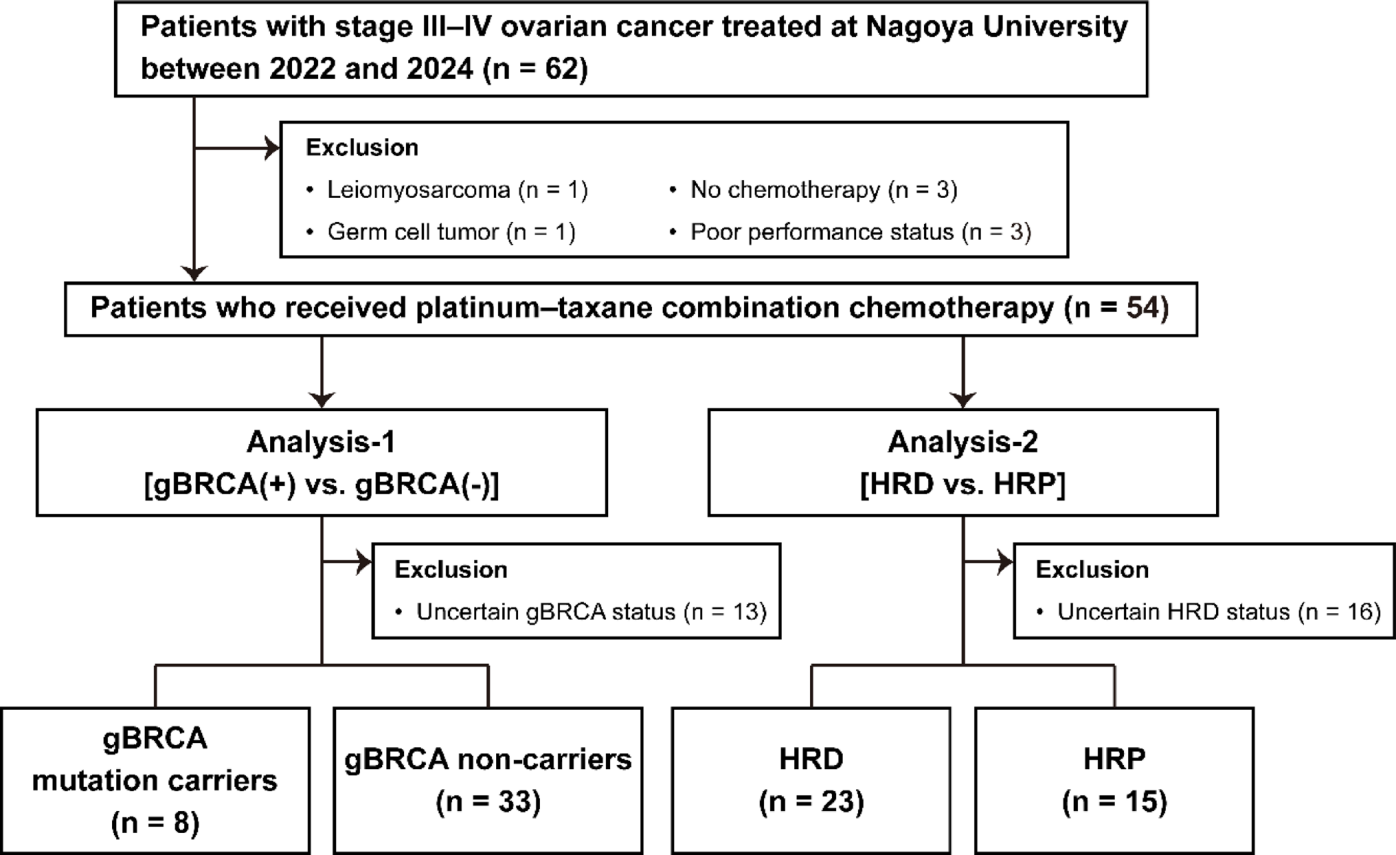
Flowchart of patient selection. Among 62 patients with stage III–IV ovarian cancer treated between 2022 and 2024, 54 patients who received platinum–taxane combination chemotherapy were included after exclusion of ineligible cases. Patients were stratified according to germline BRCA (gBRCA) or homologous recombination deficiency (HRD) status in separate analyses

Between 2022 and 2024, 62 patients with stage III–IV OC were treated at our hospital. After excluding two patients with non-epithelial cancer (leiomyosarcoma, *n* = 1; germ cell tumor, *n* = 1), three who were unwilling to receive chemotherapy, and three who received palliative chemotherapy due to poor performance status, 54 patients were included in the analysis (Fig. 1).

First, after excluding patients with uncertain gBRCA status, eight gBRCA mutation carriers (gBRCA group) were compared with 33 confirmed non-carriers (non-carrier group). In the gBRCA group, four patients had g*BRCA1* pathogenic variants and four others had g*BRCA2* pathogenic variants. In the non-carrier group, three patients had confirmed tumor *BRCA2* pathogenic variants, and one patient had a tumor *BRCA1* pathogenic variant without germline variant assessment. In addition, two patients had tumor variants of uncertain significance (*BRCA1*, *n* = 1; *BRCA2*, *n* = 1*)*, and the remaining 28 patients (84.8%) were negative for *BRCA1/2* testing. The gBRCA group had significantly higher HRD scores than the non-carrier group (*p* = 0.007; Table 1). In the non-carrier group, three patients were confirmed to have no *BRCA1/2* pathogenic variants by MyChoice^®^ analysis; however, HRD score could not be assessed. One patient could not be assigned an HRD score because only comprehensive genomic profiling was performed. In addition, two patients were confirmed to have no *BRCA1/2* pathogenic variants by BRACAnalysis^®^. Other baseline characteristics, including age, stage, histology, and treatment distribution, were comparable between groups (Table 1). Baseline laboratory tests showed that neutrophil counts were significantly higher in the gBRCA group than in the non-carrier group (5.3 vs. 4.0 × 10⁹/L, *p* = 0.042), whereas hemoglobin levels were significantly lower in the gBRCA group than in the non-carrier group (10.5 vs. 11.9 g/dL, *p* = 0.006; Table 1).

**Table 1:** Patients’ characteristics stratified by BRCA status

	gBRCA mutation carriers (*n* = 8)	gBRCA non-carriers (*n* = 33)	*p*-value
BRCA status			
g*BRCA1*, Pathogenic	4 (50.0%)	–	
g*BRCA2*, Pathogenic	4 (50.0%)	–	
t*BRCA1/2* Pathogenic	–	3 (9.1%)	
t*BRCA1/2*, VUS	–	2 (6.1%)	
Negative	–	28 (84.8%)	
HRD score			
Median (Range)	57.5 (46–84)	39 (1–92)	0.007*
Unable to analyze	–	4 (12.1%)	
NA	–	2 (6.1%)	
Age			
Median (Range)	64 (48–78)	60 (40–79)	0.500*
Stage			
III	5 (62.5%)	21 (63.6%)	1.000†
IV	3 (37.5%)	12 (36.4%)	
Histology			
HGSC	6 (75.0%)	15 (45.5%)	0.780†
Endomtrioid	1 (12.5%)	7 (21.2%)	
Clear	–	2 (6.1%)	
Adenocarcinoma	1 (12.5%)	8 (24.2%)	
Carcinoma	–	1 (3.0%)	
Treatment			
PDS plus chemotherapy	2 (25.0%)	13 (39.4%)	0.735†
NAC-IDS	5 (62.5%)	17 (51.5%)	
Chemotherapy	1 (12.5%)	3 (9.1%)	
Baseline laboratory test			
Neutrophils (×10^9^/L), Mean (Range)	5.3 (3.9–7.6)	4.0 (2.3–7.9)	0.042*
Hemoglobin (g/L), Mean (Range)	10.5 (8.6–11.8)	11.9 (7.4–14.2)	0.006*
Platelet count (×10^9^/L), Mean (Range)	382 (236–692)	321 (200–649)	0.295*

gBRCA, germline BRCA; tBRCA, tumor BRCA; VUS, variant uncertain significance; NA, not assessed; HRD, homologous recombination deficiency; HGSC, high-grade serous carcinoma; PDS, primary debulking surgery; NAC-IDS, neoadjuvant chemotherapy and interval debulking surgery; *Welch’s *t*-test; †Fisher’s exact test

**Fig. 2:**
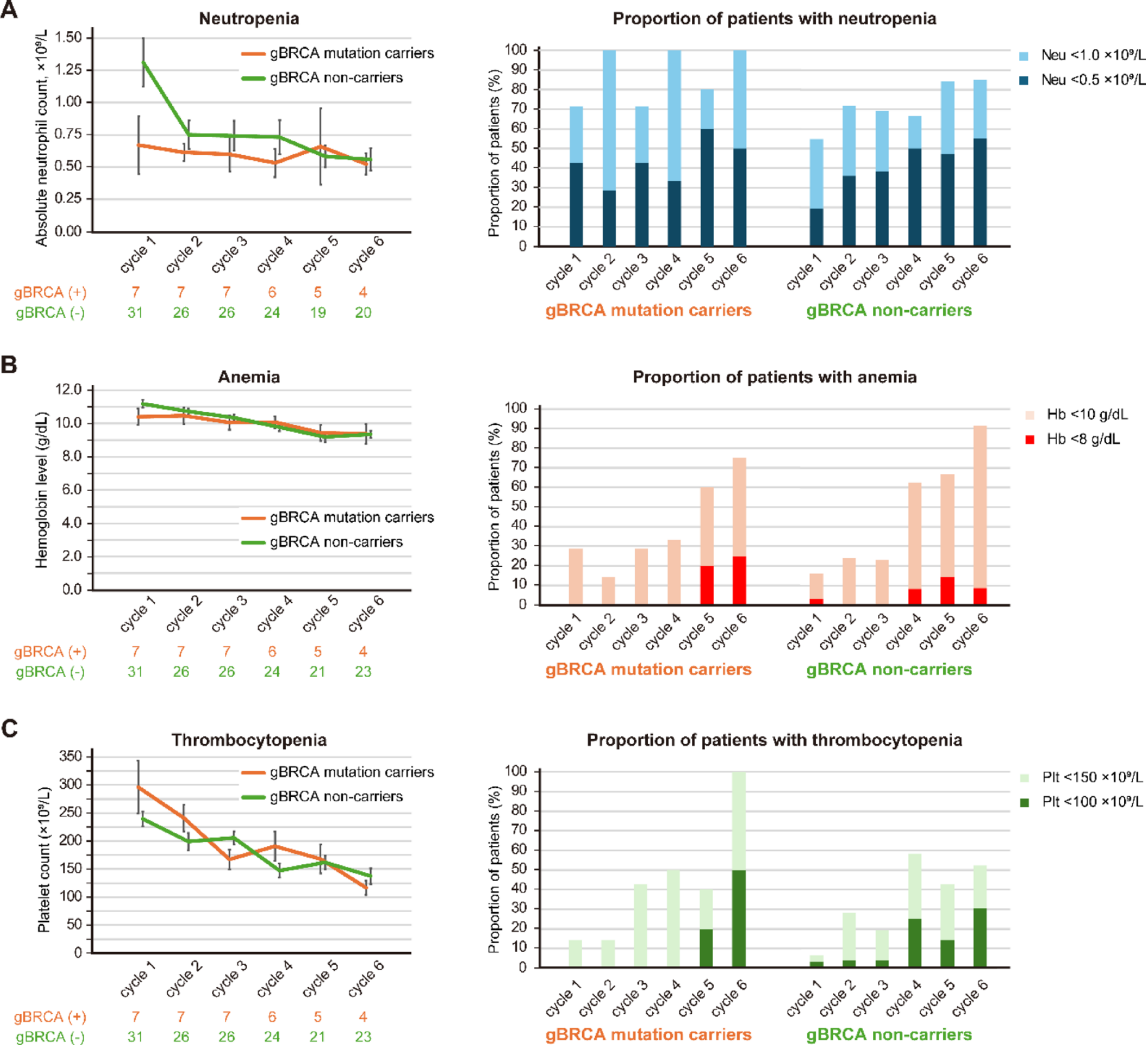
Hematologic adverse events at nadir according to gBRCA status. **A** Neutrophil counts and the proportion of patients with neutropenia (Neu < 1.0 and < 0.5 × 10^9^/L). **B** Hemoglobin levels and the proportion of patients with anemia (Hb < 10 and < 8 g/dL). **C** Platelet counts and the proportion of patients with thrombocytopenia (Plt < 150 and < 100 × 10^9^/L). Patients were classified as either gBRCA mutation carriers or non-carriers. The numbers below the x-axis indicate the number of evaluable patients at each cycle. The error bars represent the standard error of the mean. Continuous variables were compared using Welch’s t-test, and proportions were compared using Fisher’s exact test. P-values were adjusted for multiple comparisons using the Benjamini–Hochberg false discovery rate method

For the assessment of platinum–taxane combination chemotherapy-related hematologic adverse events, those at the nadir were evaluated for up to the first six cycles. In some cases, nadir assessment was not performed at the attending physician’s discretion, and these cases were excluded from the analysis. Moreover, patients who underwent IDS or had changes in their chemotherapy regimen due to disease progression were excluded from subsequent analyses. Furthermore, patients who received pegfilgrastim were excluded from neutropenia evaluation and G-CSF use for the corresponding cycle. Marked neutropenia was observed in the gBRCA group from the first cycle, whereas neutrophil counts declined progressively with increasing cycles number in the non-carrier group (Fig. 2A). At the first cycle, mean neutrophil counts were 0.67 and 1.31 in the gBRCA group and non-carrier groups, respectively, however, there was no significant difference (*p* = 0.05, adjusted *q* = 0.671). The proportions of patients with neutrophil counts < 1.0 and < 0.5 × 10^9^/L tended to be higher in the gBRCA group; the differences were not statistically significant. In addition, hemoglobin and thrombocytopenia did not significantly differ between the two groups (Fig. 2B and C). Furthermore, no statistically significant differences were observed between the gBRCA group and the non-carrier group in the frequencies of neutropenia, anemia, thrombocytopenia, G-CSF injection, dose delay, or dose reduction up to six cycles (Table 2).

**Table 2:** Frequency of toxicities up to cycle 6 in gBRCA mutation carriers vs. gBRCA non-carriers

	gBRCA mutation carriers (*n* = 8)	gBRCA non-carriers (*n* = 33)	*p*-value	q-value
Absolute neutrophil count < 1.0 × 10^9^/L	7/7 (100.0%)	27/31 (87.1%)	1.000	1.000
Absolute neutrophil count < 0.5 × 10^9^/L	5/7 (71.4%)	24/31 (77.4%)	1.000	1.000
Hemoglobin < 10 g/dL	5/7 (71.4%)	25/31 (80.6%)	0.623	1.000
Hemoglobin < 8 g/dL	1/7 (14.3%)	3/31 (9.7%)	1.000	1.000
Platelets < 150 × 10^9^/L	5/7 (71.4%)	19/31 (61.3%)	1.000	1.000
Platelets < 100 × 10^9^/L	2/7 (28.6%)	10/31 (32.3%)	1.000	1.000
G-CSF use	6/7 (85.7%)	22/31 (71.0%)	0.650	1.000
Dose delay	2/8 (25.0%)	8/33 (24.2%)	1.000	1.000
Dose reduction	3/8 (37.5%)	9/33 (27.3%)	0.692	1.000

gBRCA, germline BRCA. Patients who did not undergo any nadir check were excluded from the Neutrophil, Hemoglobin, Platelet, and G-CSF use analyses. Comparisons were performed using Fisher’s exact test, and *p*-values were adjusted for multiple comparisons using the Benjamini–Hochberg method

**Fig. 3:**
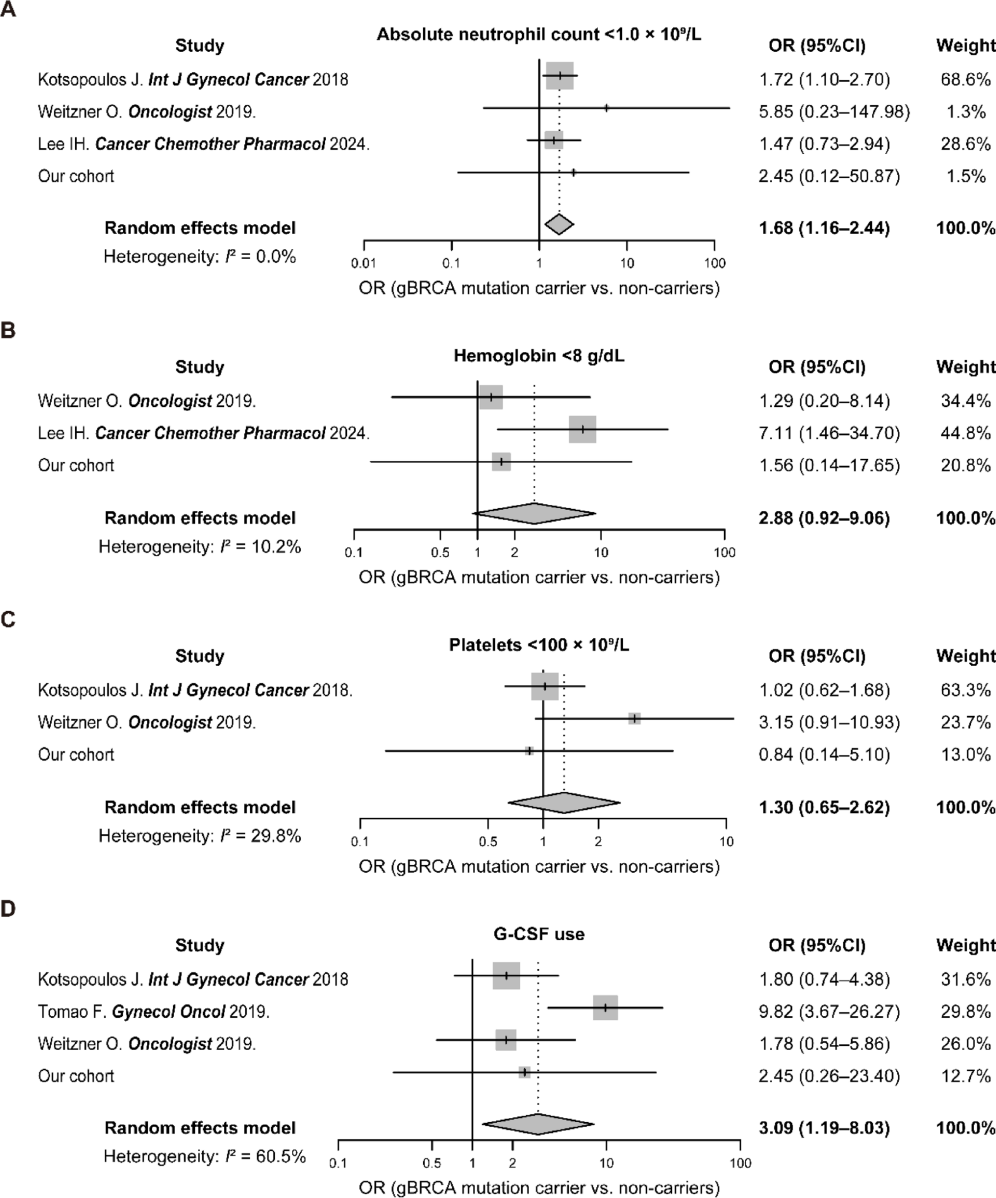
Forest plots of hematologic adverse events associated with gBRCA status. Forest plots showing the odds ratios (ORs) comparing gBRCA mutation carriers with non-carriers for **A** neutropenia defined as an absolute neutrophil count < 1.0 × 10^9^/L, **B** anemia defined as a hemoglobin level < 8 g/dL, **C** thrombocytopenia defined as a platelet count < 100 × 10^9^/L, and **D** G-CSF use. Squares represent study-specific ORs with horizontal lines indicating 95% confidence intervals (CI), and diamonds indicate pooled ORs derived from the random-effects model. OR values greater than 1.0 indicate a higher risk in gBRCA mutation carriers

Next, we constructed forest plots by integrating our results with those from previously published studies [11–14]. The frequency of neutropenia (a neutrophil count of < 1.0 × 10^9^/L) was increased in the gBRCA group compared to the non-carrier group (OR = 1.68, 95% CI 1.16–2.44; *I*^2^ = 0.0%; Fig. 3A), whereas the risk of anemia (hemoglobin levels < 8 g/dL) tended to be higher in the gBRCA group (OR = 2.88, 95% CI 0.92–9.06; *I*^2^ = 10.2%; Fig. 3B). Moreover, no significant difference was observed in the risk of thrombocytopenia (platelet counts < 100 × 10^9^/L; OR = 1.30, 95% CI 0.65–2.62; *I*^2^ = 29.8%; Fig. 3C). The risk of G-CSF use was also higher in the gBRCA group (OR = 3.09, 95% CI 1.19–8.03; *I*^2^ = 60.5%; Fig. 3D). No significant differences were observed in the rate of dose delay or reduction (Supplementary Fig.S2A, B).

**Fig. 4:**
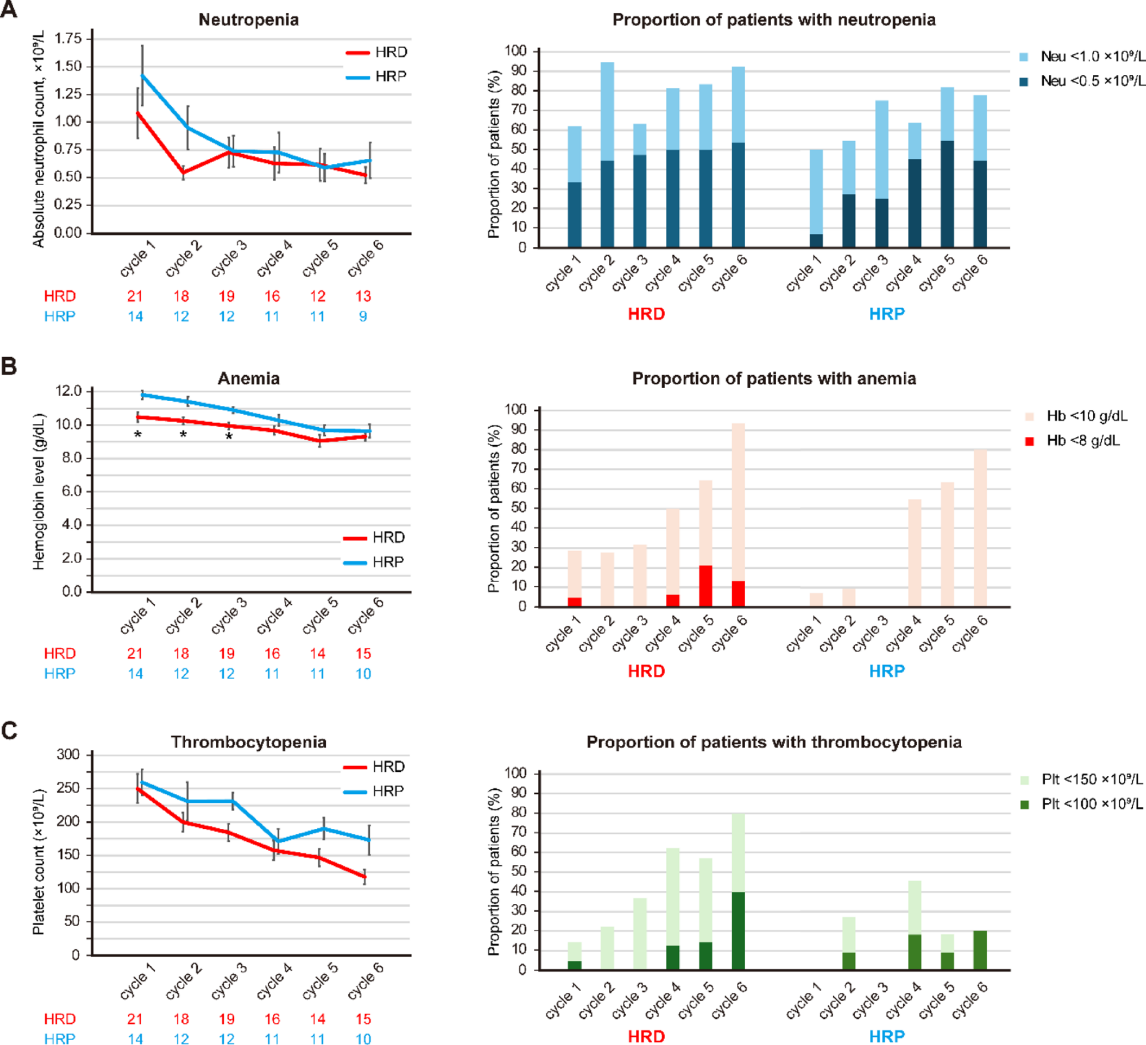
Hematologic adverse events at nadir according to HRD status. **A** Neutrophil counts and the proportion of patients with neutropenia (Neu < 1.0 and < 0.5 × 10^9^/L). **B** Hemoglobin levels and the proportion of patients with anemia (Hb < 10 and < 8 g/dL). **C** Platelet counts and the proportion of patients with thrombocytopenia (Plt < 150 and < 100 × 10^9^/L). Patients were classified as HRD or HRP. The numbers below the x-axis indicate the number of evaluable patients at each cycle. The error bars represent the standard error of the mean. Continuous variables were compared using Welch’s *t*-test, and proportions were compared using Fisher’s exact test. *P*-values were adjusted for multiple comparisons using the Benjamini–Hochberg false discovery rate method. *Adjusted *q* < 0.05

Finally, we compared the frequency of hematologic adverse events between patients with HRD-positive and HRD-negative (HRP) patients. All patients with gBRCA or tumor BRCA pathogenic variants were classified into the HRD group. As expected, HRD scores were significantly higher in the HRD group than in the HRP group (*p* < 0.001; Table 3). The distribution of histological subtypes differed significantly between the HRD and HRP groups (*p* = 0.035), and HGSC was more frequent in the HRD group (69.6%) than in the HRP group (26.7%). In addition, baseline hemoglobin levels were significantly lower in the HRD group than in the HRP group (11.1 vs. 12.7 g/dL, *p* = 0.002; Table 3). During chemotherapy, neutrophil, hemoglobin, and platelet levels tended to be lower in the HRD group than in the HRP group (Fig. 4A and C). In particular, hemoglobin levels were significantly lower in the HRD group after cycles 1, 2, and 3 (adjusted *q* = 0.027 for each cycle; Fig. 4B). However, as mentioned above, baseline hemoglobin levels were already lower in the HRD group prior to chemotherapy initiation (Table 3). The proportions of patients with neutrophil counts < 0.5 × 10^9^/L, hemoglobin < 8.0 g/dL, and platelet counts < 150 × 10^9^/L tended to be higher in the HRD group than in the HRP group; however, these differences did not reach statistical significance (Fig. 4A and C). Across up to six treatment cycles, the frequency of neutropenia < 1.0 × 10^9^/L was higher in the HRD group than in the HRP group (100% vs. 71.4%, *p* = 0.019); however, this difference was not statistically significant after adjustment for multiple comparisons (adjusted *q* = 0.172; Table 4). No other variables showed significant differences between the two groups (Table 4).

**Table 3:** Patients’ characteristics stratified by HRD status

	HRD (*n* = 23)	HRP (*n* = 15)	*p*-value
BRCA status			
g*BRCA1*, Pathogenic	4 (17.4%)	-	
g*BRCA2*, Pathogenic	4 (17.4%)	-	
t*BRCA1/2* Pathogenic	3 (13.0%)	-	
t*BRCA1/2*, VUS	2 (8.7%)	-	
Negative	10 (43.5%)	15 (100.0%)	
HRD score			
Median (Range)	61 (44–92)	25.5 (1–39)	< 0.001*
Unable to analyze	2 (8.7%)	1 (6.7%)	
Age			
Median (Range)	54 (41–78)	65 (40–76)	0.505*
Stage			
III	15 (65.2%)	8 (53.3%)	0.514†
IV	8 (34.8%)	7 (46.7%)	
Histology			
HGSC	16 (69.6%)	4 (26.7%)	0.035†
Endometrioid	4 (17.4%)	4 (26.7%)	
Clear	-	2 (13.3%)	
Adenocarcinoma	3 (13.0%)	5 (33.3%)	
Treatment			
PDS plus chemotherapy	7 (30.4%)	7 (46.7%)	0.627†
NAC-IDS	13 (56.5%)	7 (46.7%)	
Chemotherapy	3 (13.0%)	1 (6.7%)	
Baseline laboratory test			
Neutrophils (×10^9^/L), Mean (Range)	4.1 (2.4–7.6)	3.9 (2.3–7.9)	0.506*
Hemoglobin (g/L), Mean (Range)	11.1 (7.4–13.0)	12.7 (10.7–14.2)	0.002*
Platelet count (×10^9^/L), Mean (Range)	312 (200–692)	303 (228–642)	0.714*

gBRCA, germline BRCA; tBRCA, tumor BRCA; VUS, variant uncertain significance; NA, not assessed; HRD, homologous recombination deficiency; HRP, homologous recombination proficient; HGSC, high-grade serous carcinoma; PDS, primary debulking surgery; NAC-IDS, neoadjuvant chemotherapy and interval debluking surgery; *Welch’s *t-*test; †Fisher’s exact test

**Table 4:** Frequency of toxicities up to cycle 6 in HRD vs. HRP patients

	HRD (*n* = 23)	HRP (*n* = 15)	*p*-value	q-value
Absolute neutrophil count < 1.0 × 10^9^/L	21/21 (100%)	10/14 (71.4%)	0.019	0.172
Absolute neutrophil count < 0.5 × 10^9^/L	16/21 (76.2%)	10/14 (71.4%)	1.000	1.000
Hemoglobin < 10 g/dL	18/21 (85.7%)	9/14 (64.3%)	0.221	0.441
Hemoglobin < 8 g/dL	3/21 (14.3%)	0/14 (0.0%)	0.259	0.441
Platelets < 150 × 10^9^/L	15/21 (71.4%)	6/14 (42.9%)	0.159	0.441
Platelets < 100 × 10^9^/L	7/21 (33.3%)	3/14 (21.4%)	0.704	0.797
G-CSF use	18/21 (85.7%)	8/14 (57.1%)	0.112	0.441
Dose delay	7/23 (30.4%)	3/15 (20.0%)	0.709	0.797
Dose reduction	9/23 (39.1%)	3/15 (20.0%)	0.294	0.797

HRD, homologous recombination deficiency; HRP, homologous recombination proficient. Patients who did not undergo any nadir check were excluded from the Neutrophil, Hemoglobin, Platelet, and G-CSF use analyses. Comparisons were performed using Fisher’s exact test, and *p*-values were adjusted for multiple comparisons using the Benjamini–Hochberg method

To further evaluate the independent effect of HRD status, we performed an additional analysis excluding patients with gBRCA pathogenic variants. In this analysis, baseline hemoglobin levels remained significantly lower in the HRD (BRCA−) group (*p* = 0.042; Supplementary Table S2). During chemotherapy, hemoglobin levels were significantly lower after cycles 2 and 3 (adjusted *q* = 0.0208 and 0.0427; Supplementary Fig. S3), whereas neutrophil and platelet counts did not differ significantly. Supplementary Fig. S3A–C). No significant differences in hematologic adverse event frequencies were observed across six treatment cycles (Supplementary Table S3). Overall, similar trends were observed in the HRD versus HRP analysis even after exclusion of gBRCA mutation carriers.

## Discussion

In this study, we evaluated hematologic adverse events associated with platinum–taxane chemotherapy according to gBRCA and HRD status in patients with advanced OC. By integrating our institutional data with previously published reports through meta-analysis, we provide confirmatory evidence that gBRCA pathogenic variant carriers have a modestly increased risk of a neutrophil count of < 1.0 × 10^9^/L (OR = 1.68, 95% CI 1.16–2.44) and higher G-CSF use (OR = 3.09, 95% CI 1.19–8.03). Importantly, the observed differences in neutropenia or G-CSF use did not translate into meaningful downstream consequences for patients. For example, no significant differences were observed in dose reductions or treatment delays between gBRCA carriers and non-carriers, suggesting that these risks are manageable with standard supportive care and are unlikely to result in additional hospitalization or compromised oncologic outcomes. Therefore, despite the potential effect of haploinsufficiency on hematopoietic cells, our findings indicate that gBRCA carriers can safely undergo standard platinum–taxane chemotherapy without the need for prophylactic treatment modification.

Consistent with this interpretation, several studies in the setting of breast cancer have examined the association between germline BRCA pathogenic variants and chemotherapy-related hematologic toxicity. In these studies, reported differences were either absent or modest, and they were generally considered to have limited clinical relevance [15–19]. Notably, some reports suggest that BRCA1 pathogenic variants may be associated with a higher risk of hematologic toxicity compared with BRCA2 variants [15, 16]. Moreover, BRCA1 mutation carriers have been shown to experience significantly higher rates of grade 3–4 neutropenia after the first cycle of chemotherapy and increased G-CSF use [16]. However, the potential influence of specific BRCA mutation subtype, chemotherapy regimen, intensity, and patient ethnicity remains unclear. Therefore, further accumulation of cases and refined patient stratification are needed.

One possible explanation for the slightly increased susceptibility to hematologic toxicity observed in gBRCA pathogenic variant carriers is BRCA haploinsufficiency in noncancerous hematopoietic cells. Based on Knudson’s two-hit hypothesis, tumorigenesis in germline mutation carriers requires biallelic inactivation of tumor suppressor genes in cancer cells, whereas normal somatic cells already harbor a single mutated allele and retain one functional copy [20]. However, direct evidence regarding the impact of BRCA haploinsufficiency in normal hematopoietic cells remains limited. A previous report demonstrated that BRCA1 haploinsufficiency alters the expression of genes involved in cellular proliferation and differentiation in EBV-transformed lymphocytes [21]. Moreover, in vitro analyses using primary human mammary epithelial cells showed that *BRCA1* haploinsufficiency induces replication stress and genomic instability while largely preserving HR capacity [22, 23]. In this context, a review emphasized the importance of the *BRCA1* haploinsufficient phenotype in understanding *BRCA1* function [24].

In addition, our institutional cohort suggested, as a hypothesis-generating observation, that neutropenia in gBRCA carriers may occur as early as the first treatment cycle. Although chemotherapy-induced myelosuppression is generally observed over repeated treatment cycles, hematopoietic cells with BRCA haploinsufficiency may have a reduced capacity to repair DNA damage, potentially accelerating genotoxic injury from the outset of treatment. Given the very small number of gBRCA carriers in our cohort, this observation should be interpreted with caution and warrants validation in larger prospective studies.

Beyond gBRCA pathogenic variants, our institutional data provided another hypothesis-generating observation regarding HRD status. HRD status represents a broader molecular phenotype in OC, and there are several techniques for HRD testing [25]. The MyChoice^®^ HRD test evaluates HRD status using targeted sequencing of 15 h repair–related genes together with genome-wide single-nucleotide polymorphism analysis to assess genomic instability [25]. In this study, HRD-positive patients exhibited a modest tendency toward lower hemoglobin levels compared with HRP patients, and this pattern persisted even after excluding gBRCA mutation carriers. However, because baseline hemoglobin levels were already significantly lower in the HRD group prior to chemotherapy initiation, these findings should be interpreted with caution. Furthermore, the detailed algorithm used to calculate HRD scores has not been fully disclosed, and HRD represents a heterogeneous, multifactorial biological concept. Therefore, it remains challenging to delineate the precise mechanisms underlying its association with hematologic toxicity. Nevertheless, HR–related gene haploinsufficiency may represent one of the underlying reasons for this susceptibility in HRD-positive patients.

This study has several limitations. First, this is a retrospective study with a relatively small sample size, which may limit the generalizability of the findings. In particular, our institutional cohort included only eight gBRCA carriers, limiting the statistical power to detect clinically meaningful differences. Second, in the integrated analysis incorporating previously published studies, heterogeneity among reports, including differences in patient populations, ethnicity, treatment intensity, and supportive care strategies, may have influenced the observed associations. Moreover, the meta-analysis was based on a limited number of eligible studies, all of which were retrospective in design, and the total number of pooled cases remained insufficient to draw definitive conclusions. Third, this study focused exclusively on hematologic toxicity, and other adverse events were not evaluated. Given that BRCA haploinsufficiency affects all somatic cells, gBRCA pathogenic variants may also be associated with non-hematologic toxicities. In addition, patient-centered outcomes such as hospitalization rates and quality of life were not directly assessed. Fourth, the potential impact of different BRCA pathogenic variant subtypes on hematologic toxicity could not be evaluated in detail. Finally, HRD assessment was performed using heterogeneous testing platforms and scoring algorithms, which may limit the broader applicability of the HRD-related findings. In view of all these limitations, further prospective validation in larger cohorts is warranted.

In conclusion, the present study, integrating institutional data with prior reports, provides confirmatory real-world evidence that gBRCA pathogenic variant carriers have a modestly increased risk of neutropenia during platinum–taxane chemotherapy. The associations with HRD status and early-onset neutropenia remain hypothesis-generating. These differences were clinically manageable and did not necessitate changes to standard treatment strategies.

## Supplementary Information

Below is the link to the electronic supplementary material.

Supplementary Material 1

Supplementary Material 2

Supplementary Material 3

Supplementary Material 4

Supplementary Material 5

Supplementary Material 6

## Data Availability

The dataset used in this study is not publicly available due to patient privacy concerns but is available from the corresponding author upon reasonable request.
