# Supplementary figures and images for: Association of germline BRCA and homologous recombination deficiency with hematologic toxicity during platinum–taxane chemotherapy in ovarian cancer
Source: Int J Clin Oncol. 2026 May 27;31(8):1576–86. doi: 10.1007/s10147-026-03065-4 (PMC13401579; doi:10.1007/s10147-026-03065-4)

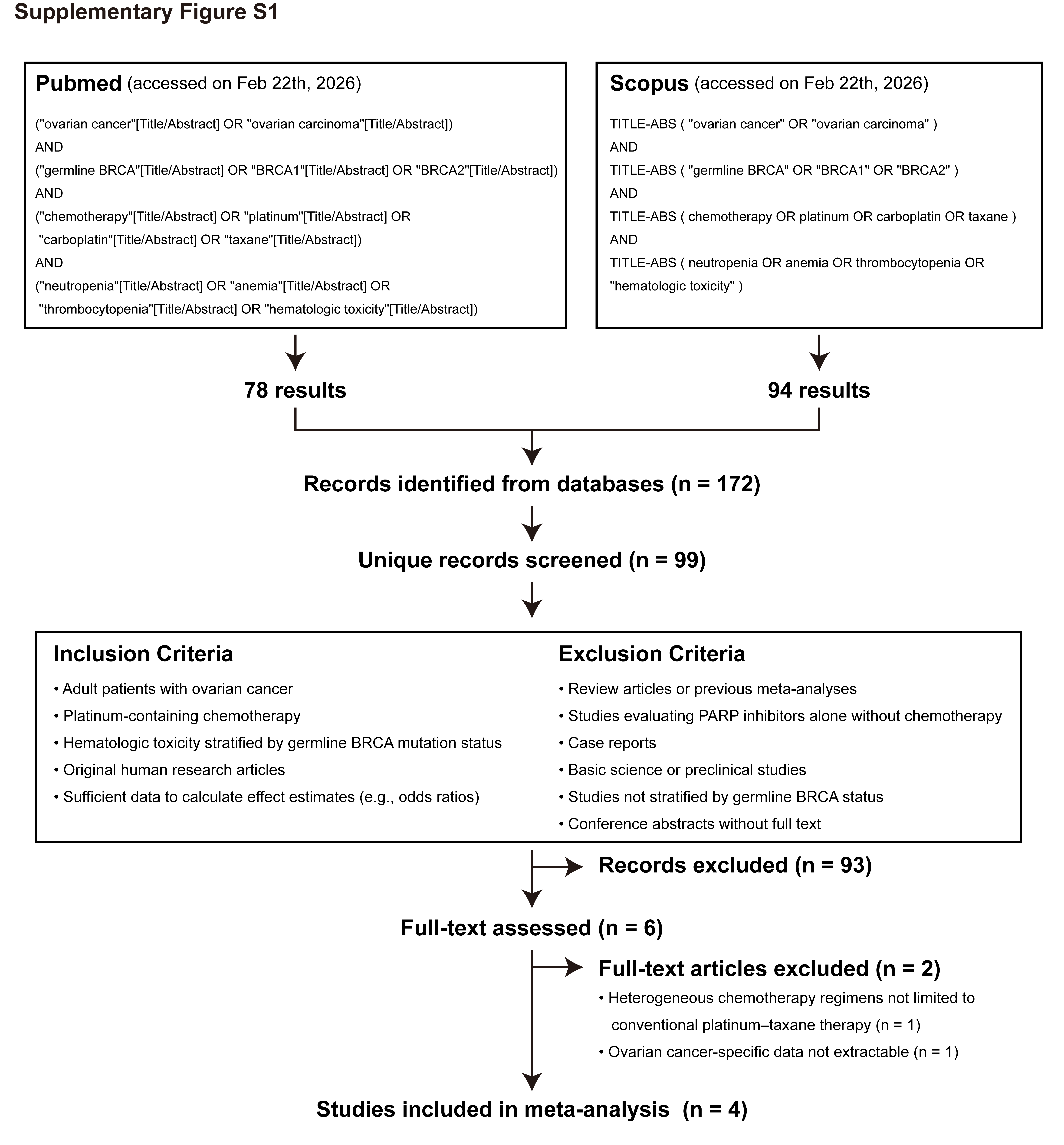

Supplement: Supplementary file 1 — Supplementary Material 1 [file 10147_2026_3065_MOESM1_ESM.png]

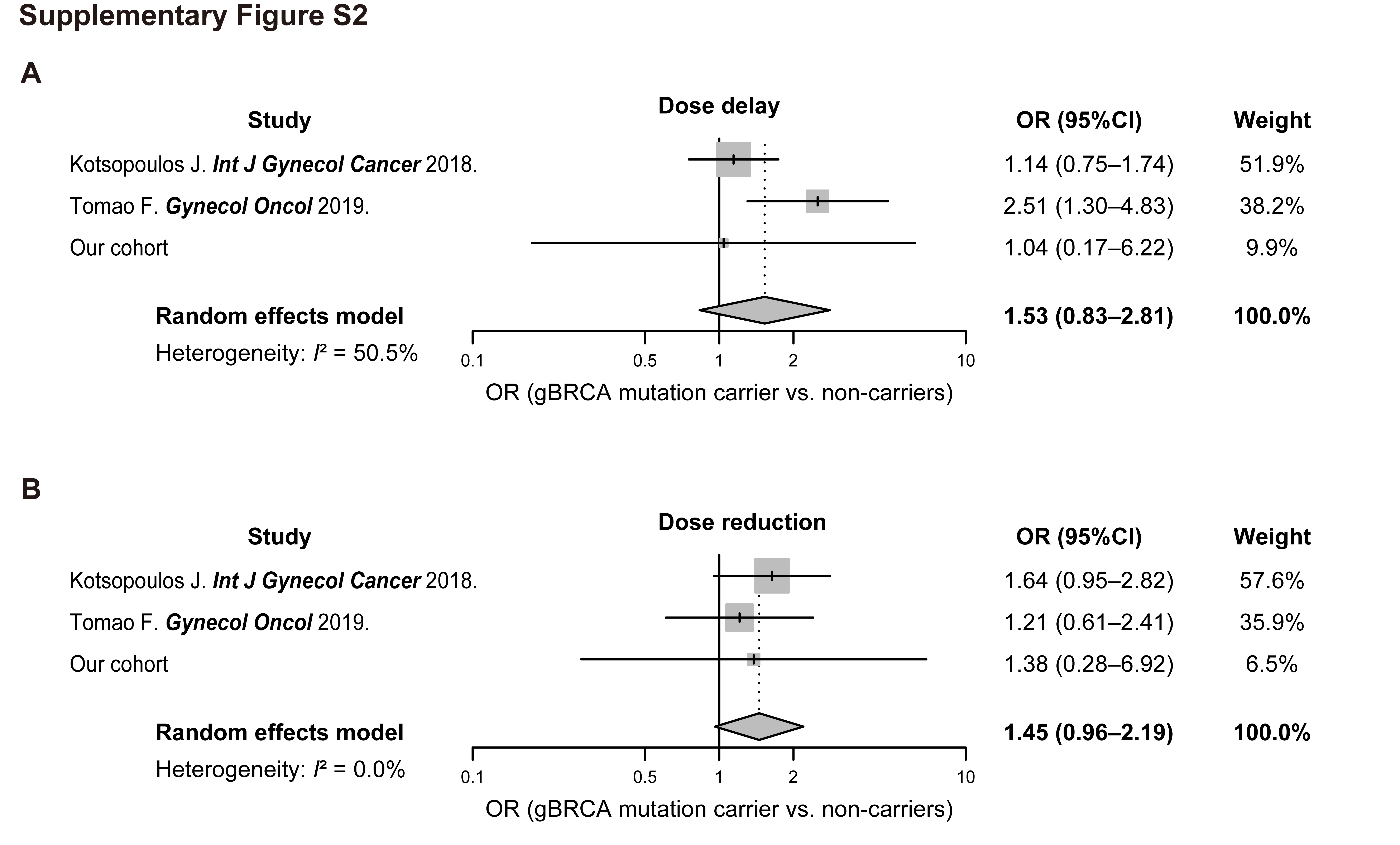

Supplement: Supplementary file 2 — Supplementary Material 2 [file 10147_2026_3065_MOESM2_ESM.png]

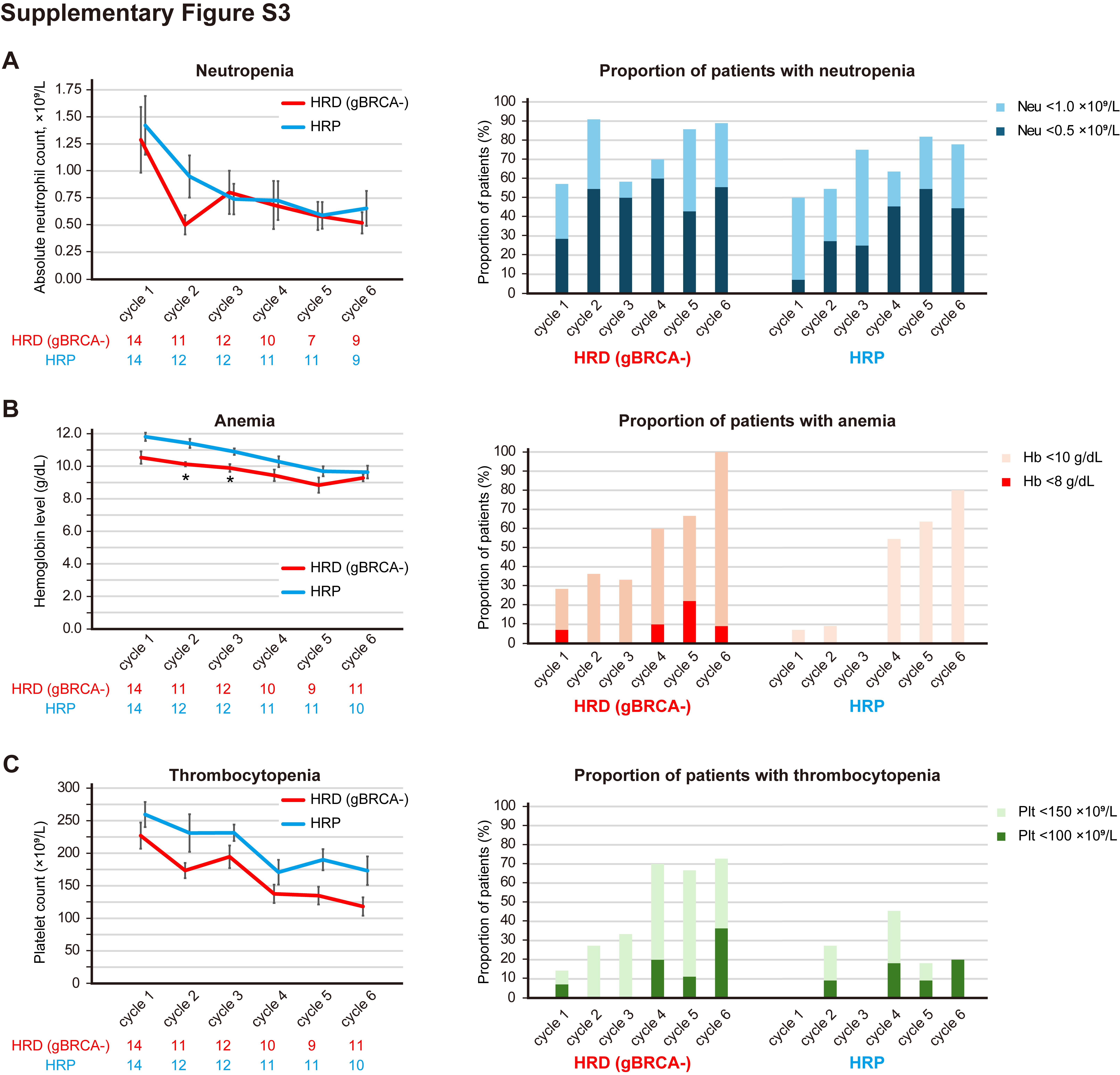

Supplement: Supplementary file 3 — Supplementary Material 3 [file 10147_2026_3065_MOESM3_ESM.png]
