# Supplementary material for: Association of germline BRCA and homologous recombination deficiency with hematologic toxicity during platinum–taxane chemotherapy in ovarian cancer
Source: Int J Clin Oncol. 2026 May 27;31(8):1576–86. doi: 10.1007/s10147-026-03065-4 (PMC13401579; doi:10.1007/s10147-026-03065-4)
Supplement: Supplementary file 5 — Supplementary Material 5 [file 10147_2026_3065_MOESM5_ESM.docx]

**Supplementary Table S2. Patients' characteristics of HRD (gBRCA-) vs. HRP patients**

|  | **HRD (gBRCA-) (n = 15)** | **HRP  (n = 15)** | ***p*-value** |
| --- | --- | --- | --- |
| **BRCA status** |  |  |  |
| t*BRCA1/2* Pathogenic | 3 (20.0%) | - |  |
| t*BRCA1/2*, VUS | 2 (13.3%) | - |  |
| Negative | - | 15 (100.0%) |  |
| **HRD score** |  |  |  |
| Median (Range) | 62 (44–92) | 25.5 (1–39) | <0.001* |
| Unable to analyze | 2 (13.3%) | 1 (6.7%) |  |
| **Age** |  |  |  |
| Median (Range) | 54 (41–76) | 65 (40–76) | 0.237* |
| **Stage** |  |  |  |
| III | 10 (66.7%) | 8 (53.3%) | 0.710† |
| IV | 5 (33.3%) | 7 (46.7%) |  |
| **Histology** |  |  |  |
| HGSC | 10 (66.7%) | 4 (26.7%) | 0.122† |
| Endometrioid | 3 (20.0%) | 4 (26.7%) |  |
| Clear | - | 2 (13.3%) |  |
| Adenocarcinoma | 2 (13.3%) | 5 (33.3%) |  |
| **Treatment** |  |  |  |
| PDS plus chemotherapy | 5 (33.3%) | 7 (46.7%) | 0.770† |
| NAC-IDS | 8 (53.3%) | 7 (46.7%) |  |
| Chemotherapy | 2 (13.3%) | 1 (6.7%) |  |
| **Baseline laboratory test** |  |  |  |
| Neutrophils (×10^9^/L), Mean (Range) | 3.9 (2.4–5.7) | 3.9 (2.3–7.9) | 0.685* |
| Hemoglobin (g/L), Mean (Range) | 11.5 (7.4–13.0) | 12.7 (10.7–14.2) | 0.042* |
| Platelet count (×10^9^/L), Mean (Range) | 321 (200–649) | 303 (228–642) | 0.862* |

gBRCA, germline BRCA; tBRCA, tumor BRCA; VUS, variant uncertain significance; NA, HRD, homologous recombination deficiency; HRP, homologous recombination proficient; HGSC, high-grade serous carcinoma; PDS, primary debulking surgery; NAC-IDS, neoadjuvant chemotherapy and interval debluking surgery; *Welch’s *t*-test; †Fisher’s exact test.
