# Supplementary material for: Association of germline BRCA and homologous recombination deficiency with hematologic toxicity during platinum–taxane chemotherapy in ovarian cancer
Source: Int J Clin Oncol. 2026 May 27;31(8):1576–86. doi: 10.1007/s10147-026-03065-4 (PMC13401579; doi:10.1007/s10147-026-03065-4)
Supplement: Supplementary file 6 — Supplementary Material 6 [file 10147_2026_3065_MOESM6_ESM.docx]

**Supplementary Table S3. Frequency of toxicities up to cycle 6 in HRD (gBRCA-) vs. HRP patients**

|  | **HRD (gBRCA-) (n = 15)** | **HRP (n = 15)** | ***p*-value** | ***q*-value** |
| --- | --- | --- | --- | --- |
| Absolute neutrophil count <1.0 × 10^9^/L | 14/14 (100%) | 10/14 (71.4%) | 0.098 | 0.567 |
| Absolute neutrophil count <0.5 × 10^9^/L | 11/14 (78.6%) | 10/14 (71.4%) | 1.000 | 1.000 |
| Hemoglobin <10 g/dL | 13/14 (92.9%) | 9/14 (64.3%) | 0.165 | 0.567 |
| Hemoglobin <8 g/dL | 2/14 (14.3%) | 0/14 (0.0%) | 0.481 | 0.722 |
| Platelets <150 × 10^9^/L | 10/14 (71.4%) | 6/14 (42.9%) | 0.251 | 0.567 |
| Platelets <100 × 10^9^/L | 5/14 (35.7%) | 3/14 (21.4%) | 0.678 | 0.767 |
| G-CSF use | 12/14 (85.7%) | 8/14 (57.1%) | 0.209 | 0.567 |
| Dose delay | 5/15 (33.3%) | 3/15 (20.0%) | 0.682 | 0.767 |
| Dose reduction | 6/15 (40.0%) | 3/15 (20.0%) | 0.427 | 0.722 |

gBRCA, germline BRCA; HRD, homologous recombination deficiency; HRP, homologous recombination proficient. Patients who did not undergo any nadir check were excluded from the Neutrophil, Hemoglobin, Platelet, and G-CSF use analyses. Comparisons were performed using Fisher’s exact test, and *p*-values were adjusted for multiple comparisons using the Benjamini–Hochberg method.
